# Derivation and Validation of Machine Learning Approaches to Predict Acute Kidney Injury after Cardiac Surgery

**DOI:** 10.3390/jcm7100322

**Published:** 2018-10-03

**Authors:** Hyung-Chul Lee, Hyun-Kyu Yoon, Karam Nam, Youn Joung Cho, Tae Kyong Kim, Won Ho Kim, Jae-Hyon Bahk

**Affiliations:** Department of Anesthesiology and Pain Medicine, Seoul National University Hospital, Seoul National University College of Medicine, Seoul 03080, Korea; azong@hanmail.net (H.-C.L.); hyunkyu18@gmail.com (H.-K.Y.); karamnam@gmail.com (K.N.); mingming7@gmail.com (Y.J.C.); ktkktk@gmail.com (T.K.K.); bahkjh@snu.ac.kr (J.-H.B.)

**Keywords:** acute kidney injury, cardiovascular surgery, machine learning

## Abstract

Machine learning approaches were introduced for better or comparable predictive ability than statistical analysis to predict postoperative outcomes. We sought to compare the performance of machine learning approaches with that of logistic regression analysis to predict acute kidney injury after cardiac surgery. We retrospectively reviewed 2010 patients who underwent open heart surgery and thoracic aortic surgery. Baseline medical condition, intraoperative anesthesia, and surgery-related data were obtained. The primary outcome was postoperative acute kidney injury (AKI) defined according to the Kidney Disease Improving Global Outcomes criteria. The following machine learning techniques were used: decision tree, random forest, extreme gradient boosting, support vector machine, neural network classifier, and deep learning. The performance of these techniques was compared with that of logistic regression analysis regarding the area under the receiver-operating characteristic curve (AUC). During the first postoperative week, AKI occurred in 770 patients (38.3%). The best performance regarding AUC was achieved by the gradient boosting machine to predict the AKI of all stages (0.78, 95% confidence interval (CI) 0.75–0.80) or stage 2 or 3 AKI. The AUC of logistic regression analysis was 0.69 (95% CI 0.66–0.72). Decision tree, random forest, and support vector machine showed similar performance to logistic regression. In our comprehensive comparison of machine learning approaches with logistic regression analysis, gradient boosting technique showed the best performance with the highest AUC and lower error rate. We developed an Internet–based risk estimator which could be used for real-time processing of patient data to estimate the risk of AKI at the end of surgery.

## 1. Introduction

Generalized linear models, such as logistic regression analysis, have been used to predict postoperative morbidity. However, the logistic regression model requires the statistical assumption of a linear relationship between the covariates and the risk of morbidity. Furthermore, the limitation of overfitting and multicollinearity of regression analysis preclude the analysis of many explanatory variables. These limitations have restricted the analysis model to select a small set of variables that are known to be clinically relevant.

Recently, the machine learning technique has been applied in areas of medicine, including detecting a specific clinical finding on medical imaging and has shown excellent performance with high sensitivity and specificity [1,2]. Additionally, there were reports about the use of machine learning techniques to predict postoperative clinical outcomes including specific morbidity or in-hospital mortality [3,4,5]. Machine learning techniques showed better performance and low error rates to predict clinical outcomes compared to the logistic regression or Cox regression analysis. However, there was also a study reporting that the machine learning technique did not show a better performance that a previous risk prediction model for in-hospital mortality [5].

Postoperative acute kidney injury (AKI) after cardiovascular surgery is known to be a relevant complication because it is associated with increased long-term mortality and development of chronic kidney disease [6,7,8]. To find a risk factor and develop a risk prediction model, previous studies reported the results of multivariable logistic regression analysis [9,10,11,12,13,14,15,16,17]. Although many risk factors and risk scores were reported by multivariable logistic regression analysis, their performance in terms of the area under the receiver operating characteristic curves (AUC) was about 0.70 to 0.83 with room for further improvement [9,10,13,14,18]. Furthermore, previous prediction models may have included an insufficient number of perioperative variables owing to overfitting and multi-collinearity of the logistic regression analysis. Additionally, the potential non-linear relationship between the covariates and the risk of outcome cannot be considered. However, machine learning techniques are relatively free of these limitations of statistical analysis and may demonstrate better performance than that of logistic regression analysis.

Therefore, we attempted to directly compare the performance and error rate of prediction with machine learning techniques with that of prediction with multivariable logistic regression analysis. We hypothesized that prediction with machine learning techniques involving many perioperative variables may demonstrate better performance and low error rate than that of logistic regression analysis. We evaluated as many machine learning techniques as possible that are currently available in the statistical software package R (version 3.4.4., R Development Core Team, Vienna, Austria) because the R software package is easily and freely accessible to investigators and many packages for machine learning approaches are currently available.

## 2. Materials and Methods

### 2.1. Study Design

This retrospective observational study was approved by the institutional review board of Seoul National University Hospital (1805-170-948). We retrospectively reviewed the electronic medical records of 2010 consecutive patients who underwent coronary artery surgery, valve replacement, or thoracic aortic surgery at our institution between 2008 and 2015. The need for informed consent was waived because of the retrospective design of the study.

### 2.2. Anesthesia, Surgical Technique

General anesthesia was maintained using a target-controlled infusion of propofol and remifentanil, or inhalational anesthetics during the study period. Standard monitoring devices were applied, including pulmonary artery catheters (Swan-Ganz CCOmbo CCO/SvO2™; Edward Lifesciences LLC, Irvine, CA, USA), in all patients.

### 2.3. Data Collection

On the basis of previous studies, data related to demographic or perioperative variables known to be related to postoperative renal dysfunction were collected (Table 1) [6,9,10,11,12,13,14,15,16,17,19,20,21,22,23]. The following perioperative clinical variables were collected: patient demographics, medical history, medication history, baseline laboratory finding, surgery type, operation time, type of anesthesia, intraoperative fluid and colloid administration, intraoperative transfusion amount, and intraoperative hemodynamic variables.

The primary outcome variable was postoperative AKI defined according to the Kidney Disease Improving Global Outcomes (KDIGO) criteria, which was determined according to the maximal change in serum creatinine level during the first seven postoperative days [6,24]. The most recent serum creatinine level measured before surgery was used as the baseline value. The detailed diagnostic criteria are shown in Table S1. We did not use the urine output criteria because previous studies suggested that different cutoffs of oliguria may be required for AKI after surgery [25,26]. We also analyzed the stage 2 or 3 AKI as secondary outcomes because stage 1 AKI may only be transient and functional and stage 2 or 3 AKI is more strongly associated with patient mortality [27]. The prediction of severe stages of AKI would be practically more important.

### 2.4. Statistical Analysis 

R software version 3.4.4. (R Development Core Team, Vienna, Austria) was used for our analysis. The following R packages for machine learning approaches were used: Tree, rpart, ROSE (Random Over-Sampling Examples), randomForest, DMwR (Data Mining with R), XGBoost (eXtreme Gradient Boosting), e1071, UBL (utility-based learning), Kernlab, nnet, neuralnet, and h2o. Tree, rpart, and ROSE packages with CART (Classification And Regression Tree) analysis were used for decision tree analysis; randomForest and DMwR were used for random forest; XGboost was used for extreme gradient boosting; e1071, UBL, and kernlab were used for support vector machine; nnet and neuralnet were used for neural network regression; and h2o was used for deep belief networks (Text S1). Seventy-two explanatory variables including variables in Table 1 were used to machine learning. Our sample was randomly divided into a training and test set with a ratio of 1:1. The coefficients of machine learning techniques were trained with the training set and tested with the test set. Our primary analysis attempted to compare the predictive accuracy of machine learning approaches with traditional analytic techniques for classification, and previous risk scores for AKI after cardiac surgery [9,10,11,12,13,14,15,16]. To evaluate and compare the predictive accuracy of prediction by machine learning techniques and logistic regression models, we calculated the areas under the receiver operating characteristics curve (AUCs) [28,29] and compared AUCs of all classifiers and models using De Long’s method [30]. We also compared the error rate, which was defined as the sum of the number of cases with false positive and false negative divided by the size of the test set. The error rates of the logistic regression model and other previous risk scores were calculated by using a cutoff where the sum of sensitivity and specificity was maximal.

For decision tree analysis, the number of terminal nodes was determined considering the scree plot showing the relationship between the tree size and coefficient of variance. We considered several decision trees with some terminal nodes that were associated with a small coefficient of variance. The final decision tree model that is clinically acceptable was chosen. The decision tree was pruned based on cross-validated error results using the complexity parameter associated with the minimal error. The ROSE package generates a synthetic balanced dataset with both over- and under-sampling and allows strengthening of the subsequent estimation of any binary outcomes [31].

The randomForest package provided a variable importance plot which shows the relative importance of the explanatory variables according to the mean decrease in accuracy or Gini. DMwR package is a technique to improve predictive ability by increasing the number of positive cases, which is called SMOTE (Synthetic Minority Over-sampling Technique). The XGBoost provides extreme and efficient gradient boosting [32,33,34]. The e1071 package was used for the support vector machine. The UBL package provides an over-sampling technique of SMOTE, which was also used to handle the class imbalance in the training set for the support vector machine [35]. The parameters of the support vector machine for classification was tuned based on balance data after SMOTE. The best parameters were determined to be a gamma of 0.1 at a cost of 10. The kernlab package provided the least square support vector machine. The neuralnet package provided the neural network classification and the number of hidden layers was defined as 6 with minimal error. The h2o deep learning package was used for deep learning.

Multivariable logistic regression analysis, including the variables in Table 1, was performed to identify independent predictors used for the development of a multivariable prediction model. To avoid multicollinearity, variables that were closely correlated with each other were excluded before being entered into the multivariable analysis. Backward stepwise variable selection was conducted using cutoff of *p* < 0.10. Previous risk scores of Palomba, Wijeysundera, Mehta, Thakar, Brown, Aronson, Fortescue, and Rhamanian et al. [9,10,11,12,13,14,15,16] were also applied to our study data and their performance was also compared with logistic models of ours, as well as other machine learning techniques. As a sensitivity analysis, logistic regression analysis without stepwise variable selection was performed to evaluate the performance.

Missing data were noted in <8% of records. We imputed the missing values according to the incidence of the missing values for each predictor. If the incidence of the missing was <2%, the missing values were substituted by the mean of continuous variables and by the mode for the incidence variable. The missing values of variables with a missing ratio of >2% and <8% were replaced using multiple imputations. Multiple imputations were performed separately in the training and test dataset. Multiple imputed training and test datasets were combined for a single run of the machine learning classifiers or logistic regression analysis.

We developed a risk estimator based on our gradient boosting model [36]. This estimator calculates the risk of developing AKI after cardiac surgery and classifies the risk into three classes of low, moderate, and high risk of AKI.

## 3. Results

A total of 2010 cases including 911 (45.3%) coronary artery bypass and 1052 (52.3%) valve replacement surgery cases were included in our analysis. During the first seven postoperative days, AKI, as determined according to the KDIGO criteria, was observed in 770 patients (38.3%) and stage 2 or 3 AKI developed in 179 patients (8.9%). The incidence of AKI was 37.3% (375/1005) for the training set and 39.3% (395/1005) for the test set. The incidences of stage 2 or 3 AKI were 9.3% (93/1005) and 8.6% (86/1005) for training and test set, respectively. Patient demographics and surgery-related variables in both training and test set are compared in Table 1.

**Figure 1:**
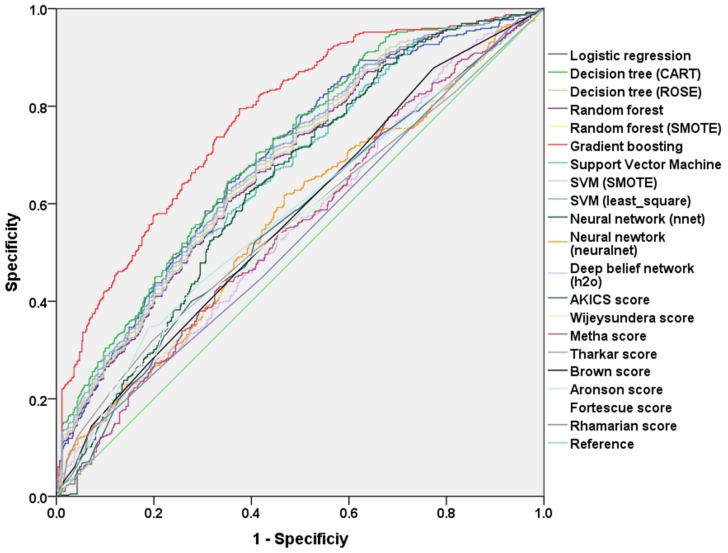
Comparison of AUC among the different machine learning models and logistic regression model. AKICS = acute kidney injury after cardiac surgery.

The error rate and AUCs of all machine techniques, logistic regression model, and risk scores to predict AKI of all stages in the test data set were compared in Table 2 and Figure 1. Extreme gradient boosting classification showed the lowest test error rate (26.0%) and the largest AUC (0.78, 95% confidence interval (CI) 0.75–0.80), which was significantly greater than AUCs of other machine learning techniques or risk scores compared (*p* < 0.001). The deep belief network classifier showed the highest test error rate (47.2%) and smallest test AUC (0.55) among all machine learning techniques compared. The error rate and AUCs to predict AKI of stage 2 or 3 in the test set were compared in Table S2. Gradient boosting classification showed lowest test error rate (8.5%) and the largest AUC (0.74). The results of multivariable logistic regression analysis with and without stepwise variable selection was shown in Table 3 and Table S3. The AUC of the multivariable logistic prediction model with stepwise variable selection was 0.69 (95% CI 0.66 to 0.72) and the model without variable selection showed similar AUC (Table 2).

**Figure 2:**
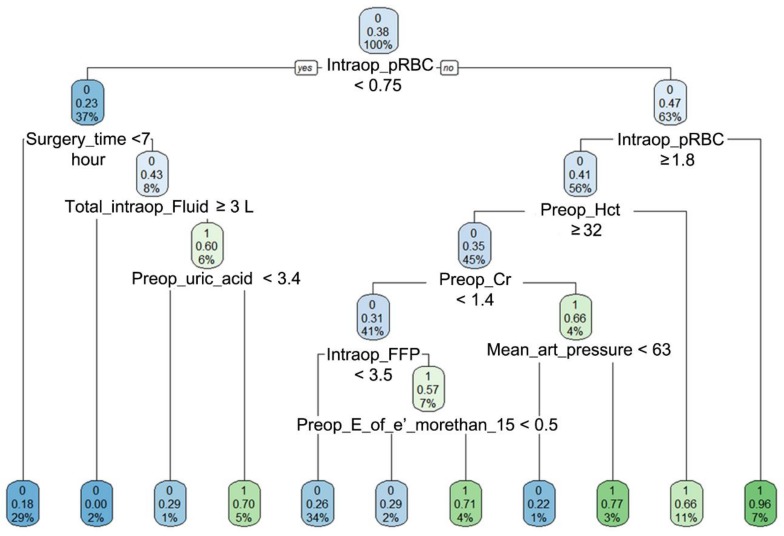
Simple decision tree model showing the classification of patients with (1) and without (0) acute kidney injury (AKI). The numbers with two decimals in each cell means the probability of developing AKI in each classification tree. The blue or green color becomes dense when it is more likely to develop acute kidney injury or not. The % number in the boxes denotes the percentage of patients with each discriminating variable from CART (Classification And Regression Tree) analysis. Intraop = intraoperative, preop = preoperative, pRBC = packed red blood cells, Hct = hematocrit, Cr = creatinine, FFP = fresh frozen plasma, E_or_e_prime = preoperative ratio of early transmitral flow velocity to early diastolic velocity of the mitral annulus.

**Figure 3:**
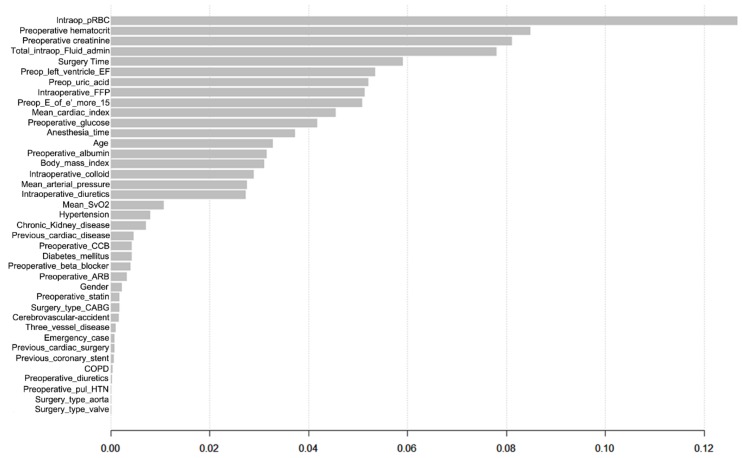
Importance matrix plot of the gradient boosting machine. This figure shows the importance of each covariates in the final model. ARB = angiotensin receptor blocker, BMI = body-mass index, CABG = coronary artery bypass graft, CCB = calcium channel blocker, CKD = chronic kidney disease, Cr = creatinine, CVA = history of cerebrovascular accident, EF = ejection fraction, E_or_e_prime = preoperative ratio of early transmitral flow velocity to early diastolic velocity of the mitral annulus, FFP = fresh frozen plasma, hct = hematocrit, HTN = hypertension, intraop = intraoperative, mean SvO2 = intraoperative mean mixed venous oxygen saturation, three_VD = three vessel coronary disease, preop = preoperative, pRBC = packed red blood cells.

Simple decision tree model showing the classification of patients with and without AKI is shown in Figure 2. The importance matrix plot of gradient boosting is shown in Figure 3 and the amount of Intraoperative red blood cells transfusion and preoperative hematocrit level were ranked the first and second. The variables of importance plot of random forest model was shown in Figure S1. The same variables were ranked first and second in terms of both mean decreases in accuracy and Gini. The matrix of classification of extreme gradient boosting was visualized in Figure S2. Figure S3 shows an example of the support vector machine classification plot.

## 4. Discussion

We compared the predictive accuracy of the prediction for AKI after cardiovascular surgery among the machine learning techniques, traditional statistical approach, and previous risk scoring models. We included currently available machine learning techniques, including decision tree, random forest, support vector machines, neural networks, and deep belief networks. Logistic regression analysis was used as the traditional approach. The results showed that extreme gradient boosting machine showed the lowest error rate and largest AUC among all techniques and risk scores, which was consistent for the prediction of stage 2 or 3 AKI. Extreme gradient boosting machine based prediction may result in significant improvement in the prediction of AKI after cardiac surgery. A risk estimator based on our gradient boosting model was developed for clinical use to determine the risk of AKI at the end of surgery.

Extreme gradient boosting showed the best predictive ability in our analysis [32,33,37]. While the random forest builds an ensemble of independent recursive partitioning tress of unlimited depth, extreme gradient boosting builds a sequential series of shallow trees, where each tree corrects for the residuals in the predictions made by all the previous tress (Figure S2). Gradient boosting uses techniques to reduce overfitting such as shrinkage and column resampling. After each step of boosting, the algorithm scales the newly added weights, which reduces the influence of each tree and allowing the model to learn better. Column resampling considers only a random subset of descriptors in building a given tree, which also fastens the training process by reducing the number of descriptors to consider [32]. It may be determined in further multicenter larger studies whether the better performance of boosting could be applied to data of other institutions or other surgical populations.

Decision tree analysis showed a similar performance to that of logistic regression model in our study. Decision trees are a hierarchical model that are comprised of decision rules based on the optimal feature cutoff values. It recursively classifies independent variables into different small groups based on the Gini impurity measure or entropy, while logistic regression analysis analyzes the interaction of included variables [38,39,40]. The odds ratio of a specific risk factor in a logistic regression model is applied to all study population rather than a single subgroup, while each branch of the decision tree may have different covariates from another branch. Variable selection in the process of decision tree is not based on probabilistic methods, which may result in overestimation of the importance of explanatory variables or may miss other potential confounders [41]. Decision trees can improve the predictive ability achieved by logistic regression models under certain circumstances. With sufficiently many terminal nodes with a low coefficient of variance, the decision tree model enables the detection of some individual cases that would have been unnoticed applying conventional logistic regression models. However, the clinical interpretation of variable selection and their cutoffs is often difficult, because the decision tree classification does not consider the clinical relevance. Decision trees are susceptible to fluctuations in the training set and are, thus, prone to overfitting and poor generalizability [4]. Additionally, decision tree models may not be practically useful if it includes too many variables. However, for the low error rate and high AUC, more classifying variables are needed.

The performance of random forest was also similar to that of logistic regression analysis in our dataset. Random forest is considered to have advantages, especially in handling electronic medical records. It is an extension to traditional decision tree classifiers [42], and attempts to mitigate the limitations of decision tree through an ensemble-based technique using multiple decision trees. Each tree is constructed from a random subset of the original training data and a random subset of a total number of variables is analyzed at each node for splitting. Random forests can minimize the problem of overfitting by taking the mode of decisions of a large number of these randomly generated trees [43]. Other advantages of random forests to analyze electronic medical records include running efficiently on large samples with thousands of input variables, the ability to accommodate different data scales, and robustness to the inclusion of irrelevant variables. There was no significant performance gain of the random forest over that of the simple decision tree in our study, which may be because the number of input variables was insufficient to demonstrate any difference in performance. 

The deep neural network model showed a good performance to predict in-hospital mortality in a previous study, although it was not superior to previous risk score [5]. Contrary to our expectations, the performance of neural network in our study was inferior to the performances of all other machine learning techniques. This may be explained because our data for learning the relationship between the covariates and risk of AKI may not be sufficient. Although the multilayer perceptron is mathematically proven to be able to approximate any nonlinear function, it requires a large amount of learning data. Therefore, the dataset of our study may not be large enough and the number of covariates was not sufficient to train the multilayer perceptron [44].

The performance of previous eight risk scoring models was poor in our test dataset [9,10,11,12,13,14,15,16]. The AUCs of these risk scores were similar possibly because similar predictors were used to construct the risk score [6], and the poor performance may be due to the small number of predictors and lack of intraoperative variables, such as transfusion amounts or hemodynamic variables. A previous study showed that the performance of the logistic regression model could be improved when we consider many perioperative variables as possible [19].

Several previous studies reported that the AUCs of machine learning techniques were not superior to previous risk scores or logistic regression models to predict postoperative mortality [5,45]. However, our study demonstrated that the AUCs of machine learning techniques could be significantly greater than the AUC of logistic regression model to predict AKI. Previous studies compared the predictive ability for in-hospital mortality in a population with a very low incidence (<1%) [5,45]. The difference in AUC or error rate may be small for an outcome with low incidence, and this small difference in performance would be difficult to be demonstrated. It seems that any difference in error rate and AUC would be more pronounced in our study sample with a postoperative AKI of higher incidence (38.3%). This could also be the reason why the SMOTE model of random forests or support vector machines did not significantly increase the AUC in our test dataset. SMOTE model increases the incidence of outcome cases and balancing the case with and without outcome variables. However, our test set already had a nearly balanced dataset for AKI.

The importance matrix plot of the gradient boosting machine shows the similar predictors that were known to be associated with the development of AKI after cardiac surgery [6,9,10,11,12,13,14,16,19,20,21,46]. However, the plot additionally gives the relative importance of each predictor, which was similar to the variance importance plot of random forest model. This analysis may help to find a new risk factor for postoperative morbidity or mortality.

Our study has several limitations. First, our analysis used only single-center data and included a relatively small number of cases and covariates. The performance of machine learning techniques might be different when they are applied to a much larger sample with a different distribution of the covariates. The external validity of our results may be limited. Furthermore, important predictors may be different according to different institutions. However, the relative performance of logistic regression and machine learning techniques would be similar to our results. Each institution may need to develop their own prediction model with the machine learning approach, by using historical data from their electronic medical records and updating the model periodically. Real-time processing of patient data would produce risk prediction for each patient after surgery. Second, machine learning techniques are often difficult to interpret the results. Inferences about the explanatory variables are more difficult than logistic regression analysis [4]. However, the gradient boosting machine and random forest provided for some interpretability through the importance matrix plot and variable importance plot. Third, it is not certain that our results could translate into improved clinical outcomes for the patients. Most of our important variables reported are not clinically modifiable and accurate risk prediction may not be followed by improved patient outcomes. However, further prospective trials may evaluate whether adjustment of potentially modifiable predictors, such as hemodynamic variables could decrease the risk of AKI [46,47,48].

## 5. Conclusions

In conclusion, our study demonstrated that the machine learning technique of extreme gradient boosting showed significantly better performance than the traditional logistic regression analysis or previous risk scores in predicting both AKI of all stages and stage 2 or 3 AKI after cardiac surgery. Gradient boosting machine may be used for real-time processing of patient data to estimate the risk of AKI after cardiac surgery at the end of surgery. Our Internet-based risk estimator may help to evaluate the risk of AKI at the end of surgery. However, prospective multicenter trials are required to validate the better prediction by gradient boosting. Further studies may apply extreme gradient boosting machine to the other important clinical outcomes after cardiac surgeries and may prospectively validate our results.

## Figures and Tables

**Table 1:** Patient characteristics and postoperative renal function in the dataset.

Variables	All	Training Set	Test Set	*p*-Value
Patient population, *n*	2010	1005	1005	
Demographic data				
Age (years)	64 (56–71)	64 (56–71)	64 (55–71)	0.884
Female (*n*)	553 (27.5)	279 (27.8)	274 (27.3)	0.803
Body-mass index (kg/m^2^)	23.8 (21.6–25.9)	23.9 (21.7–25.9)	23.7 (21.5–25.9)	0.563
Surgery type				
Coronary artery bypass (*n*)	911 (45.3)	473 (47.1)	438 (43.6)	0.117
Valvular heart surgery (*n*)	1052 (52.3)	503 (50.0)	549 (54.6)	0.060
Thoracic aortic surgery (*n*)	47 (2.3)	29 (2.9)	18 (1.8)	0.104
Emergency (*n*)	51 (2.5)	26 (2.6)	25 (2.5)	0.887
Previous cardiac surgery (*n*)	149 (7.4)	75 (7.5)	74 (7.4)	0.932
Medical history				
Hypertension (*n*)	1057 (52.6)	538 (53.5)	519 (51.6)	0.396
Diabetes mellitus (*n*)	588 (29.3)	302 (30.0)	286 (28.5)	0.433
Three vessel disease (*n*)	602 (30.0)	306 (30.4)	296 (29.5)	0.626
Previous coronary stent insertion (*n*)	235 (11.7)	118 (11.7)	117 (11.6)	0.945
Cerebrovascular accident (*n*)	228 (11.3)	101 (10.0)	127 (12.6)	0.078
COPD (*n*)	100 (5.0)	49 (4.9)	51 (5.1)	0.837
Pulmonary hypertension (*n*)	129 (6.4)	60 (6.0)	69 (6.9)	0.413
Chronic kidney disease (*n*)	121 (6.0)	57 (5.7)	64 (6.4)	0.512
Preoperative Medication				
ACEi (*n*)	114 (5.7)	58 (5.8)	56 (5.6)	0.847
ARB (*n*)	249 (12.4)	122 (12.1)	127 (12.6)	0.735
β-blocker (*n*)	289 (19.4)	199 (19.8)	190 (18.9)	0.611
Diuretics (*n*)	297 (14.8)	133 (13.2)	164 (16.3)	0.059
Calcium channel blocker (*n*)	287 (14.3)	151 (15.0)	136 (13.5)	0.339
Statins (*n*)	506 (25.2)	255 (25.4)	251 (25.0)	0.837
Aspirin (*n*)	957 (47.6)	498 (49.6)	459 (45.7)	0.090
Baseline laboratory findings				
Preoperative LVEF (%)	58 (52–63)	58 (53–63)	57 (52–63)	0.427
Hematocrit (%)	38 (34–42)	38 (34–42)	38 (34–42)	0.844
Serum creatinine (mg/dL)	0.94 (0.80–1.12)	0.93 (0.80–1.10)	0.94 (0.80–1.13)	0.613
Serum Albumin (g/dL)	4.1 (3.8–4.3)	4.1 (3.9–4.3)	4.1 (3.8–4.3)	0.183
Serum uric acid (mg/dL)	4.6 (3.7–5.6)	4.6 (3.7–5.7)	4.5 (3.6–5.5)	0.190
Blood glucose (mg/dL)	115 (96–146)	116 (96–146)	113 (96–147)	0.500
Surgery and anaesthesia details				
Operation time (h)	6.25 (5.33–7.25)	6.25 (5.41–7.27)	6.25 (5.33–7.24)	0.654
Anesthesia time (h)	7.50 (6.25–8.50)	7.50 (6.50–8.50)	7.50 (6.50–8.42)	0.608
Total intravenous anesthesia (*n*)	1858 (92.4)	937 (93.2)	921 (91.6)	0.206
Inhalational anesthesia (*n*)	152 (7.6)	68 (6.8)	84 (8.4)	0.206
Intraoperative crystalloid infusion (L)	2150 (1150–3000)	2200 (1100–3100)	2150 (1200–2950)	0.656
Intraoperative colloid use (mL)	900 (350–1500)	1000 (350–1550)	800 (350–1500)	0.067
pRBC transfusion during surgery (units)	2 (0–3)	2 (0–3)	2 (0–3)	0.725
FFP transfusion during surgery (units)	0 (0–3)	0 (0–3)	0 (0–3)	0.589
Intraoperative mean arterial pressure (mmHg)	72 (67–78)	72 (67–78)	72 (67–78)	0.974
Intraoperative mean cardiac index (L/min)	2.3 (2.1–2.7)	2.3 (2.1–2.7)	2.3 (2.1–2.7)	0.257
Intraoperative mean SvO_2_ (%)	73 (69–76)	73 (69–76)	73 (68–76)	0.207
Intraoperative diuretics use (*n*)	204 (10.1)	91 (9.1)	113 (11.2)	0.107
Postoperative renal function				
AKI according to KDIGO criteria (*n*)				0.596
Stage 1	591 (29.4)	282 (28.1)	309 (30.7)	
Stage 2	114 (5.7)	60 (6.0)	54 (5.4)	
Stage 3	65 (3.2)	33 (3.3)	32 (3.2)	
Hemodialysis dependent (*n*)	125 (6.2)	60 (6.0)	65 (6.5)	0.644
GFR at postoperative day one (ml/min/1.73m^2^)	79 (58–94)	79 (57–95)	78 (58–94)	0.864

Data are presented as median (interquartile range) or number (%). COPD = chronic obstructive pulmonary disease, ACEi = angiotensin-converting-enzyme inhibitor, AKI = acute kidney injury, ARB = angiotensin II receptor blocker, LVEF = left ventricular ejection fraction, pRBC = packed red blood cell transfusion, FFP = fresh-frozen plasma, SvO_2_ = mixed venous oxygen saturation, KDIGO = kidney disease improving global outcomes, GFR = glomerular filtration rate.

**Table 2:** Comparison of area under receiver-operating characteristic curve among the different models.

Model	Software or R Packages	Error Rate of Test Data Set	AUC in the Test Set
Machine learning techniques			
Decision tree, CART	tree, rpart	28.9%	0.71 (0.67–0.74)
ROSE decision tree	ROSE	30.6%	0.66 (0.65–0.72)
Random forest model	randomForest	30.4%	0.68 (0.64–0.71)
Random forest SMOTE model	DMwR	33.5%	0.68 (0.65–0.71)
Gradient boosting classification	XGBoost	26.0%	0.78 (0.75–0.80) *
Support vector machine, classifier	e1071	31.4%	0.67 (0.63–0.70)
Support vector machine, SMOTE model	UBL	33.3%	0.68 (0.65–0.71)
Support vector machine, least square	Kernlab	30.2%	0.69 (0.66–0.72)
Neural network classifier	nnet	38.4%	0.64 (0.61–0.68)
Neural network classifier	neuralnet	43.9%	0.57 (0.53–0.61)
Deep belief network	h2o	47.2%	0.55 (0.51–0.59)
Risk scores from logistic regression analysis			
Logistic regression model, stepwise variable selection	R	33.6%	0.69 (0.66–0.72)
Logistic regression model, without variable selection	R	32.8%	0.70 (0.68–0.73)
AKICS score	R	43.4%	0.57 (0.53–0.60)
Wijeysundera and colleagues	R	45.2%	0.55 (0.51–0.59)
Metha and colleagues	R	45.8%	0.55 (0.52–0.59)
Thakar and colleagues	R	45.3%	0.56 (0.53–0.60)
Brown and colleagues	R	43.1%	0.58 (0.54–0.61)
Aronson and colleagues	R	43.3%	0.58 (0.51–0.62)
Fortescue and colleagues	R	44.2%	0.56 (0.52–0.60)
Rhamanian and colleagues	R	47.0%	0.55 (0.52–0.58)

Error rate was defined as sum of the number of cases with false positive and false negative divided by all test set. * Significantly greater than AUC of all the other techniques, AUC = area under the receiver operating characteristic curve, CART = Classification And Regression Tree, ROSE = Random Over-Sampling Examples, SMOTE = Synthetic Minority Over-sampling Technique, DMwR = Data Mining with R, XGBoost = eXtreme Gradient Boosting, UBL = utility-based learning, AKICS = acute kidney injury following cardiac surgery.

**Table 3:** Development of multivariable logistic regression model to predict acute kidney injury using stepwise variable selection.

Variable	Beta-Coefficient	Odds Ratio	95% CI	*p*-Value
Age per 10 year	0.128	1.14	1.04–1.61	0.004
History of hypertension	0.320	1.38	1.12–1.69	0.002
Baseline chronic kidney disease	0.907	2.48	1.62–3.78	<0.001
Preoperative E/e´ > 15	0.454	1.58	1.27–1.96	<0.001
Preoperative hematocrit, %	−0.062	0.94	0.92–0.96	<0.001
Surgery time, per 1 h	0.073	1.08	1.01–1.15	0.036
Intraoperative red blood cell transfusion, unit	0.056	1.06	1.01–1.11	0.022
Intraoperative fresh frozen plasma transfusion, unit	0.085	1.09	1.03–1.15	0.001
Intraoperative diuretics use	0.630	1.88	1.36–2.60	<0.001

Multivariable logistic regression analysis was performed using all the variables in Table 1. Stepwise backward variable selection process was used for this analysis using cutoff of *p*-value of less than 0.10. Nagelkerke’s R^2^ was 0.32 and Hosmer-Lemeshow goodness-of-fit test showed good calibration (chi-square = 12.1, *p* = 0.231). CI = confidence interval, E/e´ = ratio of early transmitral flow velocity to early diastolic velocity of the mitral annulus.

## Supplementary Materials

The following are available online at http://www.mdpi.com/2077-0383/7/10/322/s1, Figure S1, Variable importance plot using the random forest model. The abbreviations were the same as the legends of Figure 3; Figure S2, Gradient boosting tree plot showing the matrix of classification. Extreme gradient boosting builds a sequential series of shallow trees; Figure S3, Support vector machine classification plot. This figure shows a simple two-dimensional visual illustration of support vector machine classification. Each triangle and circle means a binomial classification of acute kidney injury or not. The open circle or triangle means a correct classification and closed circle or triangle means an incorrect classification. This figure was drawn by Kernlab package of R; Table S1, KDIGO (Kidney Disease Improving Global Outcomes) serum creatinine diagnostic criteria of acute kidney injury; Table S2, Comparison of area under receiver-operating characteristic curve among the different models for predicting stage 2 or 3 acute kidney injury; Table S3, Results of multivariable logistic regression analysis for acute kidney injury without stepwise variable selection; Text S1, R source code to perform machine learning techniques.
