# Supplementary material for: Derivation and Validation of Machine Learning Approaches to Predict Acute Kidney Injury after Cardiac Surgery
Source: J Clin Med. 2018 Oct 3;7(10):322. doi: 10.3390/jcm7100322 (PMC6210196; doi:10.3390/jcm7100322)
Supplement: Supplementary file 1 [file jcm-07-00322-s001.pdf]

Derivation and validation of machine learning approaches to predict acute kidney injury after cardiac surgery

: Supplemental materials

Hyung-Chul Lee, **Hyun-Kyu Yoon**, **Karam Nam**, **Youn Joung Cho**, **Tae Kyong Kim**, **Won Ho Kim**, **Jae-Hyon Bahk**

Department of Anesthesiology and Pain Medicine, Seoul National University Hospital, Seoul National University College of Medicine, Seoul, Republic of Korea

List of supplemental materials

Title	Page
**Supplemental Figure S1**. Variable importance plot using random forest model.	2
**Supplemental Figure S2**. Gradient boosting tree plot showing the matrix of classification.	3
**Supplemental Figure S3**. Support vector machine classification plot.	4
**Supplemental Table S1.** KDIGO (Kidney Disease Improving Global Outcomes) serum creatinine diagnostic criteria of acute kidney injury.	5
**Supplemental Table S2.** Comparison of area under receiver-operating characteristic curve among the different models for predicting stage 2 or 3 acute kidney injury.	6
**Supplemental Table S3.** Results of multivariable logistic regression analysis for acute kidney injury without stepwise variable selection.	7
**Supplemental Text S1.** R source code to perform machine learning techniques.	9

Supplemental Figure S1. Variable importance plot using random forest model. The abbreviations were the same as the legends of Figure 3.

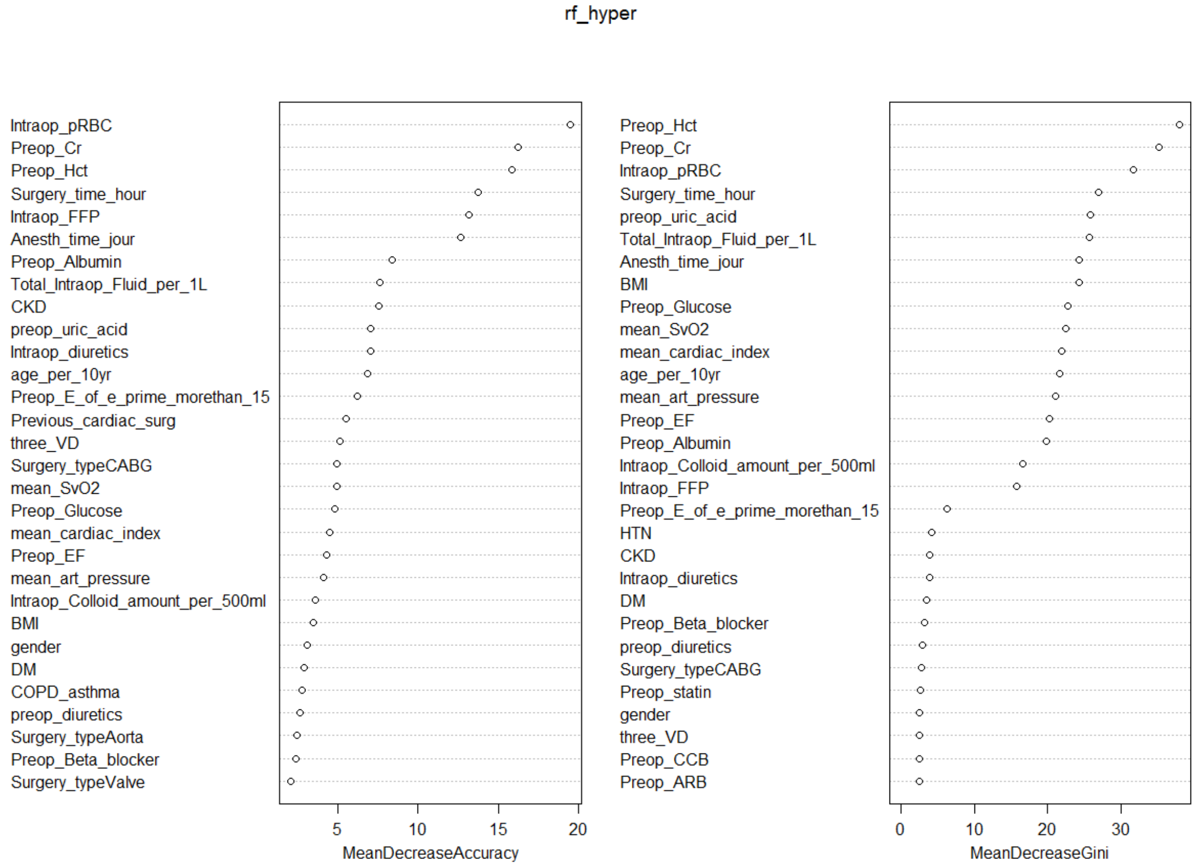

Supplemental Figure S2. Gradient boosting tree plot showing the matrix of classification. Extreme gradient boosting builds a sequential series of shallow trees.

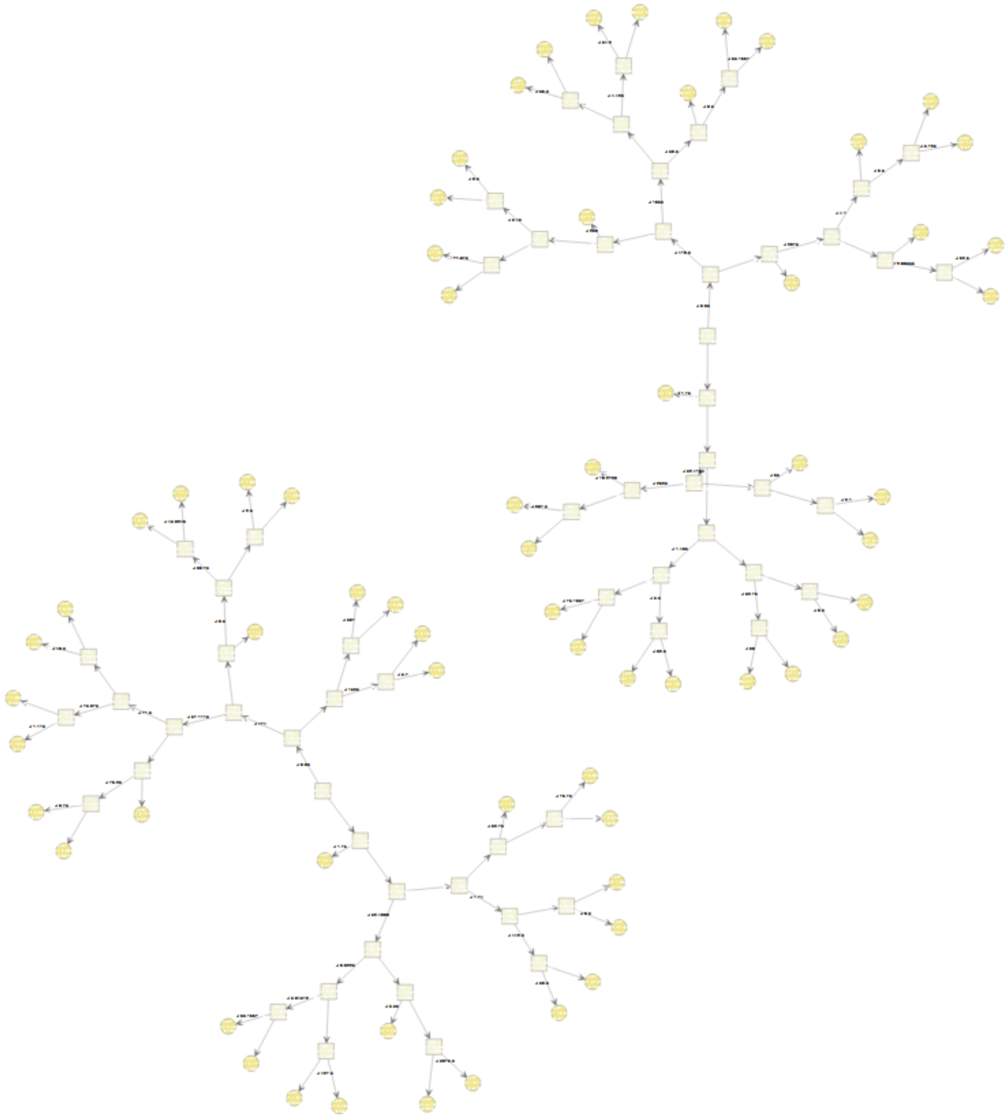

Supplemental Figure S3. Support vector machine classification plot. This figure shows a simple two-dimensional visual illustration of support vector machine classification to help understand the process of classification by support vector machine. Each triangle and circle means binomial classification of acute kidney injury or not. The open circle or triangle means correct classification and closed circle or triangle means incorrect classification. This figure was drawn by Kernlab package of software R.

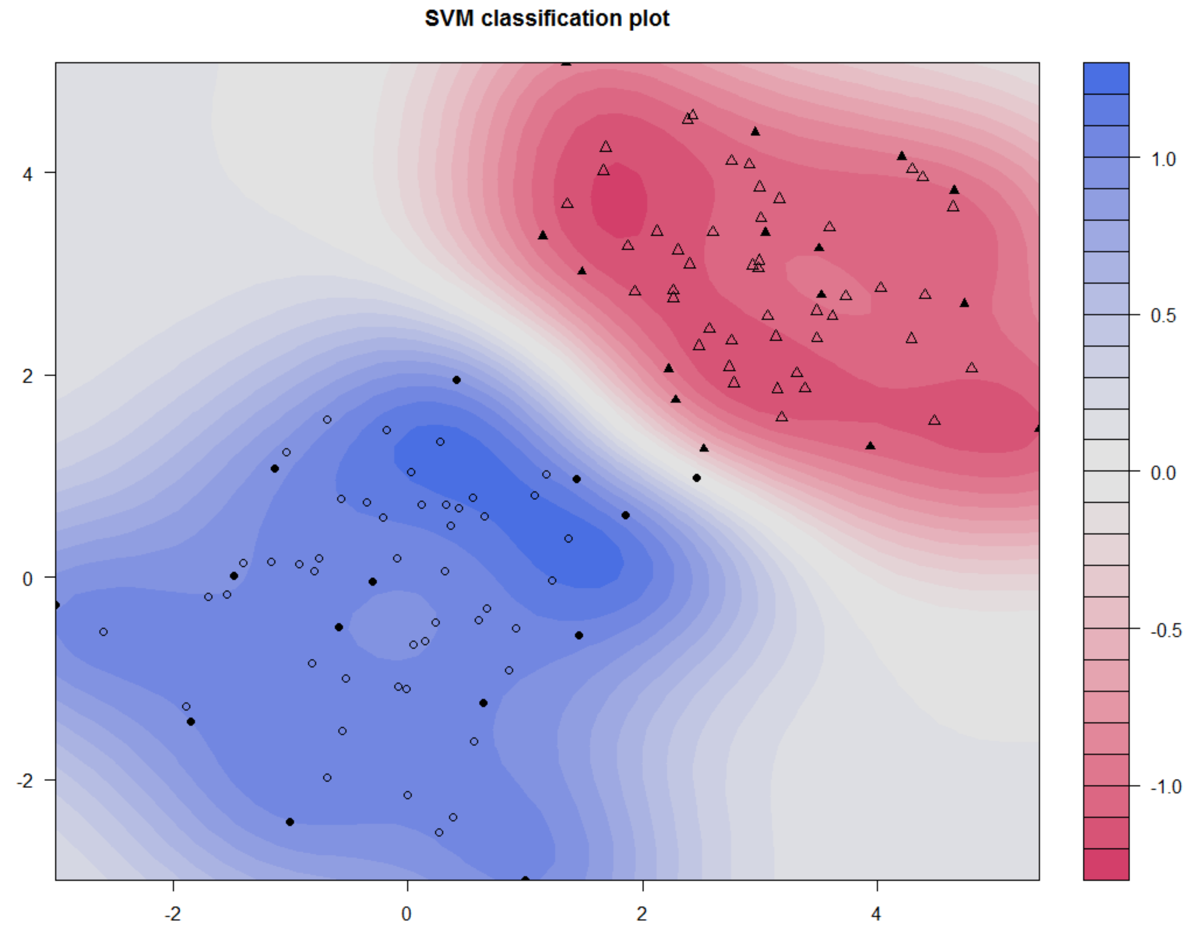

Supplemental Table S1. KDIGO (Kidney Disease Improving Global Outcomes) serum creatinine diagnostic criteria of acute kidney injury.

KDIGO criteria	Serum creatinine criteria
Stage 1	Increase in sCr by 0.3 mg/dl or increase in sCr to 1.5-1.9 times baseline within postoperative 7 days
Stage 2	Increase in sCr to 2.0-2.9 times baseline within postoperative 7 days
Stage 3	Increase in sCr to > 4.0 mg/dl or increase in sCr to 3.0 times baseline within postoperative 7 days

sCr = serum creatinine.

Supplemental Table S2. Comparison of area under receiver-operating characteristic curve among the different models for predicting stage 2 or 3 acute kidney injury.

Model	Software or R Packages	Error rate of test data set	AUC in the test set
Machine learning techniques			
Decision tree, CART	tree, rpart	14.3%	0.53 (0.50 – 0.56)
ROSE decision tree	ROSE	9.3%	0.65 (0.61 – 0.69)
Random forest model	randomForest	15.2%	0.53 (0.50 – 0.56)
Random forest SMOTE model	DMwR	13.8%	0.55 (0.51 – 0.59)
Gradient boosting classification	xgboost	8.5%	0.74 (0.70 – 0.79)*
Support vector machine, classifier	e1071	18.4%	0.53 (0.50 – 0.57)
Support vector machine, SMOTE model	UBL	12.4%	0.57 (0.53 – 0.61)
Support vector machine, least square	Kernlab	13.0%	0.54 (0.51 – 0.57)
Neural network classifier	nnet	9.9%	0.63 (0.59 – 0.67)
Neural network classifier	neuralnet	13.5%	0.54 (0.51 – 0.58)
Deep belief network	h2o	11.5%	0.57 (0.53 – 0.61)

Error rate was defined as sum of the number of cases with false positive and false negative divided by all test set.

* Significantly greater than AUC of all the other techniques.

Supplemental Table S3. Development of multivariable logistic regression model to predict acute kidney injury using stepwise variable selection.

Variable	Beta-coefficient	Odds Ratio	95% CI	p-value
Age per 10 year older	0.13	1.14	1.04 – 1.61	0.004
Female	-0.24	0.79	0.55 – 1.13	0.201
Body-mass index, kg/m^2	0.01	1.01	0.96 – 1.06	0.758
History of hypertension	0.32	1.38	1.12 – 1.69	0.002
History of diabetes mellitus	0.28	1.32	0.91 – 1.92	0.139
Baseline chronic kidney disease	0.91	2.48	1.62 – 3.78	<0.001
History of three vessel disease	-0.20	0.97	0.90 – 1.15	0.204
History of cerebrovascular accident	0.22	1.25	0.78 – 2.00	0.348
History of previous cardiac disease	0.22	1.25	0.70 – 2.24	0.452
History of previous coronary stent	-0.50	0.91	0.97 – 1.03	0.159
History of chronic obstructive pulmonary disease	-0.32	0.73	0.37 – 1.42	0.350
Preoperative E/e′ > 15	0.45	1.58	1.27 – 1.96	<0.001
Preoperative LVEF, %	0.00	1.00	0.99 – 1.02	0.783
Preoperative hematocrit, %	-0.06	0.94	0.92 – 0.96	<0.001
Preoperative creatinine, mg/dL	0.06	1.06	0.91 – 1.24	0.457
Preoperative uric acid, mg/dL	0.09	1.10	1.00 – 1.21	0.047
Preoperative albumin, g/dL	-0.30	0.74	0.53 – 1.04	0.086
Preoperative blood glucose, mg/dL	0.00	1.00	0.99 – 1.00	0.535
Preoperative beta-blocker	-0.12	0.89	0.61 – 1.29	0.535
Preoperative calcium channel blocker	0.21	1.23	0.81 – 1.87	0.334
Preoperative angiotensin receptor blocker	-0.02	0.98	0.64 – 1.52	0.937
Preoperative statin	0.18	1.20	0.84 – 1.71	0.320
Preoperative diuretics	0.26	1.30	0.82 – 2.06	0.272
Preoperative pulmonary hypertension	-0.17	0.84	0.45 – 1.56	0.580
Surgery time, per 1 hour	0.07	1.08	1.01 – 1.15	0.036
Intraoperative crystalloid administration, per 1 L	0.38	1.11	1.05 – 1.18	0.047
Intraoperative red blood cell transfusion, unit	0.06	1.06	1.01 – 1.11	0.022

Intraoperative fresh frozen plasma transfusion, unit	0.09	1.09	1.03 – 1.15	0.001
Intraoperative diuretics use	0.63	1.88	1.36 – 2.60	<0.001
Intraoperative mean cardiac index, per 0.5 L/min/m^2 decrease	0.22	1.05	1.01 – 1.09	0.045
intraoperative mean arterial pressure, per 10 mmHg decrease	0.19	1.03	1.00 – 1.04	0.048
Intraoperative mean SvO_2, per 5 % decrease	0.24	1.06	1.02 – 1.10	0.044

Multivariable logistic regression analysis was performed using all the variables in Table 1. Neither univariable screening nor stepwise variable selection process was used. Nagelkerke's R^2 was 0.38 and Hosmer-Lemeshow goodness-of-fit test showed good calibration (chi-square = 11.2, P=0.193).

CI = confidence interval, E/e′ = ratio of early transmitral flow velocity to early diastolic velocity of the mitral annulus.

Supplemental Text S1. R source code to perform machine learning techniques.

```
#------- Data loading -------#
#---- This applies to all other packages----#
aki <- read.csv("dataset.csv", header=TRUE)
n <- dim(aki)[1]
p <- dim(aki)[2]
n1 <- table(aki$AKI)[1]
n2 <- table(aki$AKI)[2]

#---- Making traing and test data sets ----#
#---- This applies to all other packages----#
set.seed(20180202)
head(aki)
ind1 <- sample(which(aki$AKI==0), round(n1/2), replace=F)
ind2 <- sample(which(aki$AKI==1), round(n2/2), replace=F)
NROW(ind1)
NROW(ind2)
tr_ind <- c(ind1,ind2)
NROW(tr_ind)
aki_tr <- aki[tr_ind,]
aki_te <- aki[-tr_ind,]

#---------- Decision Tree Classification ------------#
#--------------------------------------------------#
install.packages("tree")
library(tree)
aki_tree = tree(factor(AKI) ~., aki_tr)
summary(aki_tree)
aki_tree

#----------- Model selection for decision tree model -----------#
cv_aki.tree <- cv.tree(aki_tree)
```

```
plot(cv_aki.tree$size, cv_aki.tree$dev, type='b')

#----------- Pruning for decision tree model -----------#
dt_prun <- prune.tree(aki_tree,best=8)
plot(dt_prun)
text(dt_prun,pretty=0)

dt_prun2 = prune.misclass(aki_tree, best=8)
plot(dt_prun2)
text (dt_prun2, all = T)

dt_yht <- predict(dt_prun, newdata=aki_te[,-1],type="class")
y <- aki_te$AKI
mean(y != dt_yht)

install.packages("roc.curve")
library(roc.curve)
roc.curve(aki_te$AKI, dt_yht, plotit = F)

#---------- Decision Tree Classification ------------#
#-------- under balance case based on ROSE ----------#

install.packages("ROSE")
library(ROSE)
aki_tr_ROSE <- ovun.sample(factor(AKI) ~ ., aki_tr, method="both", seed=1, N=1500)$data
table(aki_tr_ROSE$AKI)

#----------- Training decision tree model -----------#
aki_tree_ROSE = tree(factor(AKI) ~., aki_tr_ROSE)
summary(aki_tree_ROSE)
aki_tree_ROSE

#----------- Model selection for decision tree model -----------#
```

```
cv_aki.tree_ROSE <- cv.tree(aki_tree_ROSE)
plot(cv_aki.tree_ROSE$size, cv_aki.tree_ROSE$dev, type='b')

#----------- Pruning for decision tree model -----------#
dt_prun_ROSE <- prune.tree(aki_tree_ROSE,best=25)
plot(dt_prun_ROSE)
text(dt_prun_ROSE,pretty=0)

dt_yht_ROSE <- predict(dt_prun_ROSE, newdata=aki_te[,-1],type="class")
y <- aki_te$AKI
mean(y != dt_yht_ROSE)
roc.curve(aki_te$AKI, dt_yht_ROSE, plotit = F)

#---------- Decision Tree Classification ------------#
#-------- rpart package ----------#
install.packages("rpart")
install.packages("rpart.plot")
library(rpart)
library(rpart.plot)
set.seed(21080202)
AKI_rpart = rpart(AKI ~ ., data = aki_tr, method = "class")
plotcp(AKI_rpart)
min_cp = AKI_rpart$cptable[which.min(AKI_rpart$cptable[,"xerror"]),"CP"]
AKI_rpart_prune = prune (AKI_rpart, cp = min_cp)
prp(AKI_rpart_prune)
prp(AKI_rpart_prune, type = 1)
rpart.plot(AKI_rpart_prune)
prp(AKI_rpart_prune2)
prp(AKI_rpart_prune2, type = 1)
rpart.plot(AKI_rpart_prune2)

# error rate and AUC in test set
dt_rpart_prune <- predict(AKI_rpart_prune, newdata=aki_te[,-1],type="class")
```

```
y <- aki_te$AKI
mean(y != dt_rpart_prune)
library(ROSE)
roc.curve(aki_te$AKI, dt_rpart_prune, plotit = F)

#---------------- Random Forest Regression ----------------#
#---------------------------------------------------------#
install.packages("randomForest",repos="http://cran.nexr.com")
library(randomForest)

#---- Training randomForest model ----#
hyper_tr$AKI <- as.factor(hyper_tr$AKI)
rf_hyper = randomForest(AKI~.,data=hyper_tr,mtry=round(sqrt(p-1)),importance=TRUE)
rf_hyper

#--------- Importance plot    -----------#
importance(rf_hyper)
varImpPlot(rf_hyper)

#------- prediction --------#
rf_yht <- predict(rf_hyper, newdata=hyper_te[,-1],type="class")
dim(rf_yht)

#-------- Computing error rate --------#
y <- hyper_te$AKI
dim(y)
mean(y != rf_yht)

#-------- AUC --------#
install.packages("pROC")
library(pROC)
auc <- roc(y, as.numeric(rf_yht))
print(auc)
```

```
plot(auc, ylim=c(0,1), print.thres=TRUE, main=paste('AUC:',round(auc$auc[[1]],2)))
abline(h=1,col='blue',lwd=2)
abline(h=0,col='red',lwd=2)

#--------- SMOTE more positive instances ------------#
install.packages("DMwR")
library(DMwR)
hyper_tr$AKI <- as.factor(hyper_tr$AKI)
hyper_tr_S <- SMOTE(AKI ~ ., hyper_tr, perc.over = 1980, perc.under=100)
hyper_tr_S$AKI <- as.numeric(hyper_tr_S$AKI)
hyper_tr_S$AKI <- ifelse(hyper_tr_S$AKI==1,0,1)

table(hyper_tr_S$AKI)

#----- evaluate the SMOTE performance -----#
hyper_tr_S$AKI <- as.factor(hyper_tr_S$AKI)
rf_hyper_S = randomForest(AKI~.,data=hyper_tr_S,mtry=round(sqrt(p-1)),importance=TRUE)
rf_hyper_S

#------- prediction --------#
rf_yht_S <- predict(rf_hyper_S, newdata=hyper_te[,-1],type="class")
y <- hyper_te$AKI
mean(y != rf_yht_S)

auc_S <- roc(y, as.numeric(rf_yht_S))
print(auc_S)

plot(auc_S, ylim=c(0,1), print.thres=TRUE, main=paste('AUC:',round(auc_S$auc[[1]],2)))
abline(h=1,col='blue',lwd=2)
abline(h=0,col='red',lwd=2)
```

```
#------------------- Gradient Boosting Classification -------------------#
#-------------------------------------------------------------------------#
install.packages("xgboost")
library(xgboost)

#---- Making traing and test data sets ----#
set.seed(20180202)
n1 <- table(hyper$AKI)[1]    # 3012
n2 <- table(hyper$AKI)[2]    # 151
ind1 <- sample(which(hyper$AKI==0), round(n1/2), replace=F)
ind2 <- sample(which(hyper$AKI==1), round(n2/2), replace=F)
NROW(ind1)
NROW(ind2)
tr_ind <- c(ind1,ind2)
NROW(tr_ind)
hyper_tr <- hyper[tr_ind,]
hyper_te <- hyper[-tr_ind,]
prop.table(table(hyper_tr$AKI))
prop.table(table(hyper_te$AKI))

#----- Training XGBoost model -----#
# training data
label_tr = as.vector(hyper_tr$AKI)
data_tr = as.matrix(hyper_tr[, -1])
# test data
label_te = as.vector(hyper_te$AKI)
data_te = as.matrix(hyper_te[, -1])

# Train model
bst <- xgboost(data = data_tr, label = label_tr, max_depth = 6, eta = 0.3,
                    nrounds = 2, objective = "binary:logistic")
bst1 = xgboost(data = data_tr, label = label_tr, max_depth = 6, eta = 0.3,
                    nrounds = 2, objective = "binary:logistic", eval_metric = "auc")
```

```
pred_te <- predict(bst, data_te)
pred_tr <- predict(bst, data_tr)

library(pROC)
roc_tr <- roc(label_tr, pred_tr)
roc_te <- roc(label_te, pred_te)
thres = coords(roc_tr, "best", ret = "threshold")
plot.roc(roc_tr,
         col="red",
         print.auc=TRUE,
         max.auc.polygon=TRUE,
         print.thres=TRUE,
         auc.polygon=TRUE)
plot.roc(roc_te,
         col="red",
         print.auc=TRUE,
         max.auc.polygon=TRUE,
         print.thres=TRUE,
         auc.polygon=TRUE)

print(paste("Threshold: ", thres))
pred_tr <- ifelse (pred_tr > thres, 1, 0)
print(paste("Train Error: ", mean(pred_tr != label_tr)))
pred_te <- ifelse (pred_te > thres, 1, 0)
print(paste("Test Error: ", mean(pred_te != label_te)))
table(label_te, prediction)

#------ View feature importance/influence from the learnt model ------#
importance_matrix <- xgb.importance(model = bst)
print(importance_matrix)
xgb.plot.importance(importance_matrix = importance_matrix)

#---- View the trees from your model ----#
```

```
xgb.dump(bst, with_stats = T)

#----- Plot the trees from your model -----#
install.packages("DiagrammeR")
library(DiagrammeR)
xgb.plot.tree(model = bst)

#------ Cross validation ---------#
dtrain <- xgb.DMatrix(data_tr,label=label_tr)
cv_res = xgb.cv(data = dtrain, nfold = 5, nround = 2, nthread = 2,
                max_depth = 3, eta = 1, objective = "binary:logistic", eval_metric = "auc")


#-------- Support Vector Machine Regression ---------#
#--------------------------------------------------#
install.packages("e1071")
library(e1071)

#------- Tuning the parameters of SVM   --------#
svm_tune <- tune(svm, factor(AKI)~., data=aki_tr, ranges = list(cost =c(1,10), gamma=c(0.1,0.5)))
summary(svm_tune)

svm_tune$best.parameters
svm_after_tune <- svm(factor(AKI)~.,data=aki_tr, scale=F, gamma=0.1,   cost=1)
#default: kernel="radial", gamma=1/n, cost=10, epsilon=0.1
summary(svm_after_tune)
svm_after_tune$fitted

y_pred <- predict(svm_after_tune, aki_te[,-1])
mean(aki_te[,1]!= y_pred)

table(aki_te[,1], y_pred)
```

```
1- sum(diag(table(aki_te[,1], y_pred)))/(dim (aki_te)[1])

install.packages("pROC")
library(pROC)
auc <- roc(aki_te$AKI, as.numeric(y_pred))
print(auc)

plot(auc, ylim=c(0,1), print.thres=TRUE, main=paste('AUC:',round(auc$auc[[1]],2)))
abline(h=1,col='blue',lwd=2)
abline(h=0,col='red',lwd=2)

#------ Apply SMOTE to aki_tr data ------#
install.packages("UBL")
library(UBL)

aki_tr$AKI <- as.factor(aki_tr$AKI)
aki_tr_s <- SmoteClassif(AKI ~ ., aki_tr, C.perc = "balance")
aki_tr_s <- SmoteClassif(AKI ~ ., aki_tr, C.perc = "balance", dist="HOEM")
aki_tr_s <- SmoteClassif(AKI ~ ., aki_tr, C.perc = "balance", dist="HVDM")
aki_tr_s
aki_tr_s$AKI <- as.numeric(aki_tr_s$AKI)
table(aki_tr_s$AKI)
aki_tr_s$AKI <- ifelse(aki_tr_s$AKI==1,0,1)

table(aki_tr_s$AKI)
prop.table(table(aki_tr_s$AKI))

#------- Tuning the parameters of SVM based on balanced data after SMOTE    --------#
svm_tune_s  <-  tune(svm,  factor(AKI)~.,  data=aki_tr_s,  ranges  =  list(cost  =c(1,10),
gamma=c(0.1,0.5)))
summary(svm_tune_s)
svm_tune_s$best.parameters
svm_after_tune_s <- svm(factor(AKI)~.,data=aki_tr_s, scale=F, gamma=0.1,    cost=10)
```

```
summary(svm_after_tune_s)
svm_after_tune_s$fitted

# Apply to test set
y_pred_s <- predict(svm_after_tune_s, aki_te[,-1])
mean(aki_te[,1]!= y_pred_s)
table(aki_te[,1], y_pred_s)
1- sum(diag(table(aki_te[,1], y_pred_s)))/(dim(aki_te)[1])

install.packages("pROC")
library(pROC)
auc_s <- roc(aki_te$AKI, as.numeric(y_pred_s))
print(auc_s)

plot(auc_s, ylim=c(0,1), print.thres=TRUE, main=paste('AUC:',round(auc_s$auc[[1]],2)))
abline(h=1,col='blue',lwd=2)
abline(h=0,col='red',lwd=2)

#-- Least Squares Support Vector Machine Classification (kernlab)--#
#----------------------------------------------------------------------#
install.packages("kernlab")
library(kernlab)

#---- Making traing and test data sets ----#
set.seed(20180202)
n1 <- table(aki$AKI)[1]
n2 <- table(aki$AKI)[2]
ind1 <- sample(which(aki$AKI==0), round(n1/2), replace=F)
ind2 <- sample(which(aki$AKI==1), round(n2/2), replace=F)
NROW(ind1)
NROW(ind2)
tr_ind <- c(ind1,ind2)
NROW(tr_ind)
```

```
aki_tr <- aki[tr_ind,]
aki_te <- aki[-tr_ind,]
prop.table(table(aki_tr$AKI))
prop.table(table(aki_te$AKI))

#------- Training LS-SVM model    --------#
lssvm <- lssvm(factor(AKI)~., scaled=F, reduced = F, cross=0,kernel = "rbfdot", kpar=list(sigma=0.1), tau=0.1, data=aki_tr)
summary(lssvm)
fitted(lssvm); alpha(lssvm); b(lssvm)
ypred_lss <- predict(lssvm, aki_te[,-1])
mean(aki_te[,1]!= ypred_lss)

table(aki_te[,1], ypred_lss)
1- sum(diag(table(aki_te[,1], ypred_lss)))/(dim(aki_te)[1])

install.packages("pROC")
library(pROC)
auc_s <- roc(aki_te$AKI, as.numeric(y_pred_lss))
print(auc_s)

#-------- Neural Network Regression -----------------#
#--------------------------------------------------------#
install.packages("nnet")
library(nnet)

#------- Tuning neural network (nnet): determing the number of hidden nodes for test data ----
#
err<-c()
for(h_size in seq(6, 8, by = 1))
{ nn <- nnet(factor(AKI)~., data=hyper_tr, size = h_size, decay = 0, trace=F,linout=F)
py <- predict(nn, hyper_te[,-1], type = "class")
ty <- hyper_te$AKI
```

```
err[h_size] <- mean(ty != py)}
best_size <- which.min(err)

#------- Predicting neural network (nnet) for test data after tuning --------#
nn_model <-nnet(factor(AKI)~., data=hyper_tr, size = best_size, decay = 0, trace=F,linout=F)
ypred <- predict(nn_model, hyper_te[,-1], type = "class")
mean(hyper_te$AKI != ypred)
table(hyper_te$AKI, ypred)
1- sum(diag(table(hyper_te$AKI, ypred)))/(dim(hyper_te)[1])

#-------- Neural Network Regression -----------------#
#---------------------------------------------------#
install.packages("neuralnet")
library(neuralnet)

#--Tuning neural network (neuralnet): determing the number of hidden nodes for test data ---#
err_cv <- c()
for(h_size in seq(6, 8, by = 1))
{neural_cv
<-neuralnet(hyper_tr$AKI~
Emergency+Surgery_typeCABG+Surgery_typeAorta+Surgery_typeValve+age_per_10yr+ge
nder+BMI+HTN+DM+COPD_asthma+CVA+CKD+Pul_HTN+three_VD+Previous_PCI_ste
nt+Previous_cardiac_surg+Preop_EF+Preop_E_of_e_prime_morethan_15+Preop_Hct+Preo
p_Cr+Preop_Albumin+preop_uric_acid+Preop_Glucose+Preop_ARB+Preop_ACEi+Preop_
CCB+Preop_Beta_blocker+Preop_statin+preop_diuretics+Anesth_technique_TIVA_vs_inhal
ational+Surgery_time_hour+Anesth_time_hour+mean_art_pressure+mean_SvO2+mean_car
diac_index+Total_Intraop_Fluid_per_1L+Intraop_Colloid_amount_per_500ml+Intraop_pRB
C+Intraop_FFP+Intraop_diuretics, data=hyper_tr[,-1], hidden = h_size,
algorithm = "rprop+", err.fct="ce",linear.output=FALSE)
   ypr_cv <- compute(neural_cv, hyper_te[,-1])
   ypred_cv <- ifelse(ypr_cv$net.result > 0.5, 1, 0)
   err_cv[h_size] <- mean(hyper_te$AKI != ypred_cv)
}
```

```
best_size <- which.min(err_cv)

#------- Prediction based neural network (neuralnet) after tuning --------#
model_form = as.formula(paste("hyper_tr$AKI~ ",paste(names(hyper_tr[,-1]),collapse='+')))
neural_f <- neuralnet(hyper_tr$AKI~
Emergency+Surgery_typeCABG+Surgery_typeAorta+Surgery_typeValve+age_per_10yr+ge
nder+BMI+HTN+DM+COPD_asthma+CVA+CKD+Pul_HTN+three_VD+Previous_PCI_ste
nt+Previous_cardiac_surg+Preop_EF+Preop_E_of_e_prime_morethan_15+Preop_Hct+Preo
p_Cr+Preop_Albumin+preop_uric_acid+Preop_Glucose+Preop_ARB+Preop_ACEi+Preop_
CCB+Preop_Beta_blocker+Preop_statin+preop_diuretics+Anesth_technique_TIVA_vs_inhal
ational+Surgery_time_hour+Anesth_time_hour+mean_art_pressure+mean_SvO2+mean_car
diac_index+Total_Intraop_Fluid_per_1L+Intraop_Colloid_amount_per_500ml+Intraop_pRB
C+Intraop_FFP+Intraop_diuretics, data=hyper_tr[,-1], hidden = 6,
algorithm = "rprop+", err.fct="ce",linear.output=FALSE)

ypr <- compute(neural_f, hyper_te[,-1])
ypred <- ifelse(ypr$net.result > 0.5, 1, 0)

mean(hyper_te$AKI != ypred)
table(hyper_te$AKI, ypred)

#--------- Deep Belief Network based Classification -------------#
#---------------------------------------------------------------#
install.packages("h2o")
library(h2o)
h2o.init()

#------- Data loading -------#
hyper <- read.csv('dataset.csv', header=T)
head(hyper,3)
n <- dim(hyper)[1]
p <- dim(hyper)[2]
print(table(hyper$AKI))
```

```
print(prop.table(table(hyper$AKI)))

#--- 10-fold cross validation method without pretraining -----#
h1 <- 100; h2 <- 100; h3 <- 100;
dnn_cv <- h2o.deeplearning(x=2:26,y=1,activation="Tanh",#activation="TanhWithDropout",
                           #initial_weight_distribution = "Normal",
                           mini_batch_size=6,epoch=100,
                           hidden=c(h1,h2,h3),training_frame=hyper_tr,
                           nfolds=10, balance_classes = FALSE)
acc <- dnn_cv@model$cross_validation_metrics_summary[1,1]
acc

#------------ Training DNN model --------------#
#----- Pretraining using autoencoder -----#

ae_pretr <- h2o.deeplearning(x = 2:26, training_frame = hyper_tr,
                             model_id = "cc_autoencoder", ignore_const_cols =
FALSE,
                             activation = "Tanh",
                             hidden = c(h1,h2,h3), autoencoder = TRUE)

#----- Fine tuning using BP -----#
dnn_fit1 <- h2o.deeplearning(x = 2:26, y = 1, training_frame = hyper_tr,
                             ignore_const_cols = FALSE, hidden = c(h1,h2,h3),
                             pretrained_autoencoder = "cc_autoencoder")

#------------ Prediction based on DNN model --------------#
h2o.performance(dnn_fit1,hyper_te)
h2o.confusionMatrix(dnn_fit1,hyper_te)

#--------- Training DNN model using BP without pretraining -------#
dnn_fit2 <- h2o.deeplearning(x = 2:26, y = 1, training_frame = hyper_tr,
                           ignore_const_cols = FALSE, hidden = c(h1,h2,h3))
```

```
h2o.performance(dnn_fit2,hyper_te)
h2o.confusionMatrix(dnn_fit2,hyper_te)
```
